# Treating benign paroxysmal positional vertigo in acute traumatic brain injury: a prospective, randomised clinical trial assessing safety, feasibility, and efficacy

**DOI:** 10.1136/bmjno-2023-000598

**Published:** 2024-05-28

**Authors:** Rebecca M Smith, Caroline Burgess, Jenna Beattie, Abby Newdick, Vassilios Tahtis, Bithi Sahu, John F Golding, Jonathan Marsden, Barry M Seemungal

**Affiliations:** 1 Centre for Vestibular Neurology, Department of Brain Sciences, Imperial College London, London, UK; 2 School of Population Health & Environmental Studies, King's College London, London, UK; 3 Occupational Therapy, Imperial College Healthcare NHS Trust, London, UK; 4 Physiotherapy, St George's Healthcare NHS Trust, London, UK; 5 Occupational Therapy, King's College Hospital NHS Foundation Trust, London, London, UK; 6 Psychology, University of Westminster, London, UK; 7 School of Health Professions, University of Plymouth, Plymouth, Plymouth, UK

**Keywords:** NEUROOTOLOGY, VERTIGO, REHABILITATION, RANDOMISED TRIALS, TRAUMATIC BRAIN INJURY

## Abstract

**Background:**

Benign paroxysmal positional vertigo (BPPV) affects approximately half of acute, moderate-severe traumatic brain injury (TBI) patients. To date, there have been no rigorous studies of BPPV assessment or treatment in this cohort. We aimed to determine the safety, practicability, and efficacy of therapist-led BPPV management in acute TBI and the feasibility of a larger effectiveness trial.

**Methods:**

This was a multi-centre, three-arm, parallel-groups, randomised, feasibility trial. Recruitment was via convenience sampling. The main inclusion criteria were age over 18 years and a confirmed, non-penetrating, acute TBI. BPPV-positive patients were randomly allocated to one of three interventions (repositioning manoeuvres, Brandt–Daroff exercises or advice) using minimisation criteria. Outcome assessors were blinded to the intervention.

**Results:**

Of 2014 patients screened for inclusion, 180 were assessed for BPPV. Of those assessed, 34% (62/180) had BPPV, and 58 patients received an intervention. Therapist-led interventions were delivered safely and accurately according to intervention monitoring criteria. Resolution of BPPV was observed in 35/58 (60%) patients. The resolution rate was highest following repositioning manoeuvres (78%), followed by the advice (53%) and Brandt–Daroff interventions (42%). 10 patients experienced recurrence. This was observed more frequently in those with skull fractures and bilateral or mixed BPPV.

**Conclusions:**

Overall, the results provide strong evidence for the feasibility of a future trial. Therapist-led management of BPPV in acute TBI was safe and practicable. Repositioning manoeuvres seemingly yielded a superior treatment effect. However, given the high recurrence rate of post-traumatic BPPV, the optimal time to treat according to patients’ specific recurrence risk requires further investigation.

**Trial registration:**

ISRCTN91943864, https://doi.org/10.1186/ISRCTN91943864.

**WHAT IS ALREADY KNOWN ON THIS TOPIC**

- There are no prospective data on the safety, practicability or effectiveness of BPPV assessment and treatment in patients with acute traumatic brain injury.

**WHAT THIS STUDY ADDS**

- This first prospective, randomised, multi-centre trial of adults with acute post-traumatic BPPV found therapist-led assessment and treatment of BPPV was safe and practicable. Repositioning manoeuvres were more efficacious for BPPV resolution than standard exercises or advice. A high recurrence rate was noted.

**HOW THIS STUDY MIGHT AFFECT RESEARCH, PRACTICE OR POLICY**

- The results provide the basis to definitively establish (a) the effectiveness of repositioning manoeuvres and (b) the optimal timing of treatment delivery.

## Introduction

Traumatic brain injury (TBI) is the most common cause of morbidity and mortality in under 40-year-olds in the UK.^1^ Imbalance and dizziness secondary to vestibular dysfunction, involving damage to peripheral and/or central vestibular structures, affect most patients with moderate-to-severe TBI.^2^ Persistent vestibular dysfunction is linked to physical and psychosocial morbidity,^3^ including delayed return-to-work^4^ and falls.^5^ Critically, falls causing unintentional trauma are linked to excess mortality in community-dwelling TBI survivors.^6^

Benign paroxysmal positional vertigo (BPPV) is the most frequent cause of peripheral vestibular dysfunction in acute TBI^2 7^ and is a treatable risk factor for falls in idiopathic BPPV.^8^ However, current evidence indicates that routine assessment and treatment of BPPV in acute TBI is uncommon,^9 10^ seemingly due to a variety of knowledge and role-based barriers.^10^ Notably, previous qualitative work demonstrated that healthcare professionals had concerns regarding the assessment and treatment procedures for BPPV in the acute TBI cohort, while post-traumatic dizziness was viewed by some as self-limiting and non-specific.^10^ These concerns also included practical considerations such as patients’ pain, cognition and how to complete procedures in those with spinal and limb trauma. Healthcare professionals were also apprehensive about patients’ tolerability and response to treatment.^10^ Currently, there are no patient- or healthcare professional-derived data on the tolerability, practicability or acceptability of BPPV assessment and treatment procedures in acute TBI. This would be critical for the implementation of any changes to practice. Furthermore, there are no prospective data on the effectiveness of treatment, via repositioning manoeuvres, in post-traumatic BPPV. Indeed, despite the frequency of BPPV in the TBI population, the latest clinical guidelines for early head injury management do not mention the need for vestibular, or more specifically BPPV, assessment or treatment.^11^

Due to a paucity of data relating to trauma settings, treatment of post-traumatic BPPV is based on guidelines developed specifically for idiopathic BPPV.^12^ Post-traumatic BPPV differs from idiopathic BPPV in that it is linked to (1) vestibular agnosia (an attenuated vertigo sensation),^13^ (2) frequent recurrence and multi-canal involvement^14^ and (3) co-occurrence with other vestibular diagnoses.^2^ Previous animal model research has suggested post-traumatic BPPV is linked to acute disruption of the inner ear ultrastructure, which persists up to 3 months following injury,^15^ thus potentially rendering a single manoeuvre delivered acutely ineffective. Further, studies conducted in idiopathic BPPV have noted links between inflammatory markers and the presence and recurrence of BPPV.^16–18^ Although there is little data regarding inflammatory markers in post-traumatic BPPV, there is literature on persisting pathological inflammation post-TBI.^19^ It is thus possible that a post-TBI inflammatory state may affect responses to BPPV treatment.

Thus, given the lack of evidence regarding the assessment and treatment of post-traumatic BPPV, we aimed to determine the safety, practicability and efficacy of managing this common condition, as well as the feasibility of conducting a future effectiveness trial.

## Design and methods

### Design

This was a multi-centre, three-arm, parallel-group, randomised, feasibility trial, which recruited across three major trauma centres in London, UK, from February 2020 to September 2021. Patients consenting to participate, and who then tested positive for BPPV on examination, completed outcome measures pre-treatment and at 4 and 12 weeks post-treatment. The published research protocol contains more methodological details.^20^ Data regarding clinicians’ and patients’ views on the acceptability of trial procedures, critical for the future implementation of BPPV treatment in acute TBI, will be reported separately.

Ethical permission for the study was granted by the East of England Research Ethics Committee (19/EE/0052). A trial steering committee, which included clinicians, patient experts and lay members, oversaw the conduct of the trial.

### Participants

Participants were medically stable adults aged ≥18 years, on an in-patient trauma or outlying rehabilitation ward, with a confirmed non-penetrating traumatic brain injury. Exclusion criteria included cervical spine instability precluding BPPV assessment, medical instability, pregnancy, a current prescription of phenytoin (which can produce central positional nystagmus) and Glasgow Coma Score of <14. Consenting patients, irrespective of complaints of vertigo, were assessed for posterior and horizontal canal BPPV by trained trauma ward therapists. Patients with atypical nystagmus were not included in the study. Similar to other recent clinical trials,^21^ posterior and horizontal BPPV were diagnosed according to Barany Society criteria (i.e., for posterior canal BPPV, a positive diagnosis was made on the presence of torsional nystagmus with the upper pole of the eye beating towards the lower ear combined with vertical nystagmus beating upwards, following a latency of one or a few seconds) via clinical examination findings by trained therapists.^12 22^ Therapists received standardised training from the lead researcher. Further details regarding the training delivered to ward therapists have been documented previously.^23^

### Randomisation

Patients with BPPV were sequentially randomised, using an online platform, to one of three interventions (re-positioning manoeuvres, Brandt–Daroff exercises or advice). Currently, routine practice in trauma centres does not include ward-based acute vestibular assessment and treatment.^10^ Thus, the advice arm constituted no change to current clinical practice, that is, comprised of the ‘usual’ or ‘standard’ care for participants. Additionally, there are currently no prospective, randomised treatment studies in acute TBI-related BPPV; thus, there are no data to guide the constituents of an effective intervention or the feasibility of an intervention. The lack of evidence is of critical importance, since the latest clinical guidelines,^11^ which follow evidence-based medicine, also do not advise routine assessment and treatment of BPPV in acute TBI. Minimisation criteria were used to ensure a similar distribution of patient-specific factors between intervention groups, including age ≤40 years vs >40 years), TBI severity (highest Glasgow coma scale at 24 hours following GCS ≥9 vs GCS <9) and ability to complete balance measures (Yes vs No). Outcome assessors were blinded to group allocation.

### Interventions

Interventions were delivered during participants’ in-patient admission. Regardless of treatment group allocation, therapists were asked to re-assess patients following the treatment and before discharge. Participants who remained BPPV positive at the trial end point (12-week follow-up) were offered further treatment regardless of group allocation.

Re-positioning manoeuvres were delivered by trained ward therapists and followed clinical practice guidelines.^12^ Therapists were able to complete up to three repositioning manoeuvres in one session (depending on patients’ symptoms and tolerability) and up to three sessions in total over subsequent days where applicable. As per the published protocol, therapists were trained in using the Epley and Semont manoeuvres for posterior canal BPPV, and the log roll manoeuvre for horizontal canal BPPV.^20^ Following treatment, therapists re-assessed patients during their in-patient stay to evaluate their response to treatment, including the possibility of canal conversion. Participants were not instructed to complete self-manoeuvres at home.

Participants allocated to the Brandt–Daroff group received two therapist-supervised sessions during their in-patient stay. Patients were instructed to continue with the exercises twice daily for a total of 2 weeks and to keep a record of their adherence.

Participants in the advice group received two inpatient sessions in which they received verbal advice on standing and moving safely and on performing natural head and body movements. A written advice sheet was also provided (online supplemental 1). This level of advice represents standard care in many UK major trauma centres.^10 23^

10.1136/bmjno-2023-000598.supp1 Supplementary data

### Trial progression criteria, adverse events and intervention monitoring

Trial progression criteria were set to determine the appropriateness of moving forwards to a more definitive randomised controlled trial. Criteria included ≥60% of patients screened being eligible for inclusion, ≥30% of eligible patients consenting and ≤40% dropout rate. Serious adverse events (vomiting and falls), treatment duration (ie, the length of time recorded by clinicians spent delivering treatment to patients in the hospital), clinician confidence with the intervention (using a visual analogue scale) and intervention fidelity were recorded. To ascertain intervention fidelity, videos (see online supplemental video) of all three interventions across all sites were moderated by an independent clinical specialist vestibular physiotherapist with >15 years of experience. The moderator rated the interventions against criteria that were set using clinical practice guidelines.^12^

10.1136/bmjno-2023-000598.supp2 Supplementary video

### Measures

Baseline demographics were recorded, including age, gender, mechanism and severity of the head injury, functional status and falls. Primary outcome measures related to feasibility objectives: number of eligible patients, recruitment and retention rates, adverse events and variability in intervention fidelity. Secondary outcomes were collected at baseline, 4 weeks post-treatment and 12 weeks post-treatment and comprised the diagnostic tests for both posterior and horizontal canal BPPV to ascertain resolution and/or recurrence, self-reported measures and objective measures. Recurrence of BPPV was defined as a positive Dix–Hallpike or supine head roll test at the 4- or 12-week follow-up, preceded by a documented negative test (either following treatment or at 4-week follow-up). Self-reported measures included those relating to dizziness and balance (Dizziness Handicap Inventory,^24^ Activities-specific Balance Confidence (ABC) scale^25^ and the UCLA Dizziness questionnaire),^26^ mood (Hospital Anxiety and Depression scale),^27^ health outcome (EQ-5D))^28^ and TBI recovery measures (Glasgow Coma Outcome Score^29^ and Quality of Life after Brain Injury).^30^ Bedside objective balance measures included the modified clinical test of sensory interaction in balance^31^ and the modified dynamic gait index; an eight-item measure comprising tasks such as 6 m usual speed ambulation without and with horizontal and vertical head turns.^32^ The complete range of measures and the time points at which they were completed are noted in online supplemental table 1).

### Statistical analysis

A power calculation was not performed before this study as the primary aim was to assess feasibility. Normality of data was assessed using graphical and statistical methods. For normally distributed data, the means and SD are reported. For non-normally distributed data, medians and interquartile ranges (IQR) are reported. χ² or Fisher’s exact tests were used to analyse data pertaining to skull fractures (using participants with and without BPPV) and BPPV resolution. Analysis of skull fracture data was completed using data from hospital CT reports. Odds ratios (OR) were used to evaluate categorical frequency. Mann–Whitney U test (non-normally distributed data) and two-way mixed analysis of variance, where group refers to the between-subject factors (manoeuvres vs Brandt–Daroff vs advice) and time refers to the within-subject factors (baseline vs follow-up), analysed treatment effects. Analyses were performed using R Statistical Software (2022.12.0; R Core Team 2021).

## Results

### Recruitment and feasibility objectives

**Figure 1:**
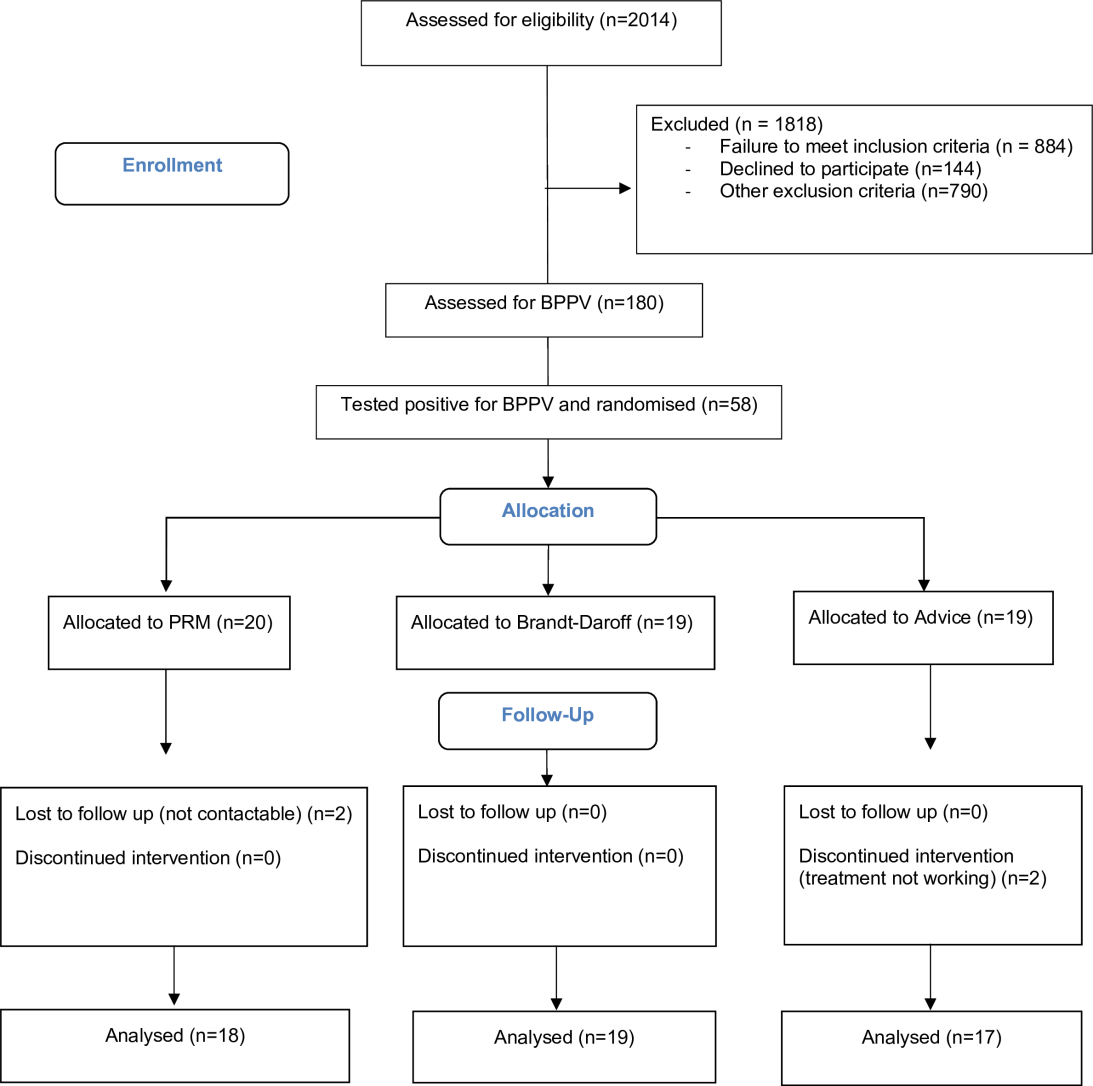
Participant flow diagram

Results are reported in line with CONSORT feasibility guidelines.^33^ Of the 2014 patients screened, 1818 were either excluded or declined to participate. An estimation of total head injuries during the recruitment period totalled 2400. 196 of the 547 eligible patients (36%) consented to participate (figure 1). 16/196 (8%) patients could not be assessed for BPPV due to early discharge. Of 180 patients assessed for BPPV, 62 (34%) tested positive. On average, patients were assessed for BPPV at 6 (IQR 8) days post-injury. Four patients were discharged before randomisation and treatment and thus were not enrolled in the study. Hence 58 patients were enrolled and randomised.

The trial drop-out rate was 7%. Other pre-set trial progression criteria including the number of patients eligible for inclusion and the number consenting were met indicating appropriateness to move towards a full trial. No serious adverse events relating to assessment or treatment were recorded. Six adverse events of vomiting in five patients were recorded, occurring either post-diagnostic or repositioning manoeuvre. Adverse events were equally distributed among treatment groups (online supplemental table 2).

### Baseline characteristics and symptom reporting

The baseline characteristics of the whole sample are summarised in table 1. The demographics of our cohort are similar to participants in other TBI studies; the sample was relatively young (mean 53.5 years) with a male predominance (67%). The majority of the sample (78%) had a moderate-severe TBI.^34^ 30/58 (51.7%) patients had unilateral BPPV, while 23/58 (39.6%) had bilateral BPPV. 5/58 (8.7%) had a mixed posterior and horizontal canal BPPV. The whole sample’s gait speed was 0.68 m/s. Patients had relatively few comorbidities. 11/58 (18%) of patients had a previous history of hypertension, 4/58 (6%) had suffered prior falls, 1/58 (1.7%) had a pre-injury diagnosis of migraine and 9/58 (15%) reported dizziness in the previous 6 months. Patients with bilateral BPPV had a significantly slower gait speed than those with unilateral BPPV (95% CI −0.03 to –0.51, p=0.04; online supplemental table 3). Notably, 19% of patients (11/58) denied dizziness before diagnostic testing. An attenuated sensation of dizziness, despite manifesting peripheral vestibular activation, that is, vestibular agnosia, was also found in this cohort; that is, 5/58 (9%) participants denied any illusory self-motion during diagnostic testing, and a further 6/58 (10%) reported only mild dizziness. 9/58 (15%) of patients received prochlorperazine, while no patients received betahistine during their acute admission.

**Table 1:** Baseline characteristics of the 58 included participants with BPPV

Clinical characteristic	Manoeuvres (20)	Brandt–Daroff (19)	Advice (19)
Age, mean (SD)	53.3 (15.27)	52.68 (18.29)	54.57 (20.48)
Sex, n (%)			
Male	14 (70%)	15 (79%)	10 (53%)
Female	6 (30%)	4 (21%)	9 (47%)
Injury details			
GCS, median (IQR)	14 (1.5)	14 (2.5)	15 (1)
Moderate-severe TBI, n (%)	14 (70%)	11 (57%)	15 (79%)
Mechanism – falls, n (%)	8 (40%)	9 (47%)	8 (42%)
Mechanism – RTA, n (%)	7 (35%)	7 (37%)	8 (42%)
Skull fractures, n (%)	13 (65%)	13 (68%)	10 (53%)
BPPV details			
Unilateral BPPV, n (%)	8 (40%)	13 (68%)	9 (47%)
Bilateral BPPV, n (%)	9 (45%)	4 (21%)	10 (53%)
Mixed BPPV, n (%)	3 (15%)	2 (11%)	0 (0%)
Clinical variables			
DHI, median (IQR)	36 (46)	15 (2)	22 (3)
FAC, independent, n (%)	10 (50%)	12 (63%)	12 (63%)
Pre-existing dizziness, n (%)	2 (10%)	4 (21%)	3 (16%)
Vestibular agnosia, n (%)	2 (10%)	1 (5%)	2 (11%)

Mixed BPPV, Posterior and horizontal BPPV; DHI, Dizziness handicap inventory; FAC, functional ambulation category; RTA, road traffic accident; GCS (at the scene), Glasgow Coma Score.

### BPPV resolution, recurrence and link to skull fracture

**Figure 2:**
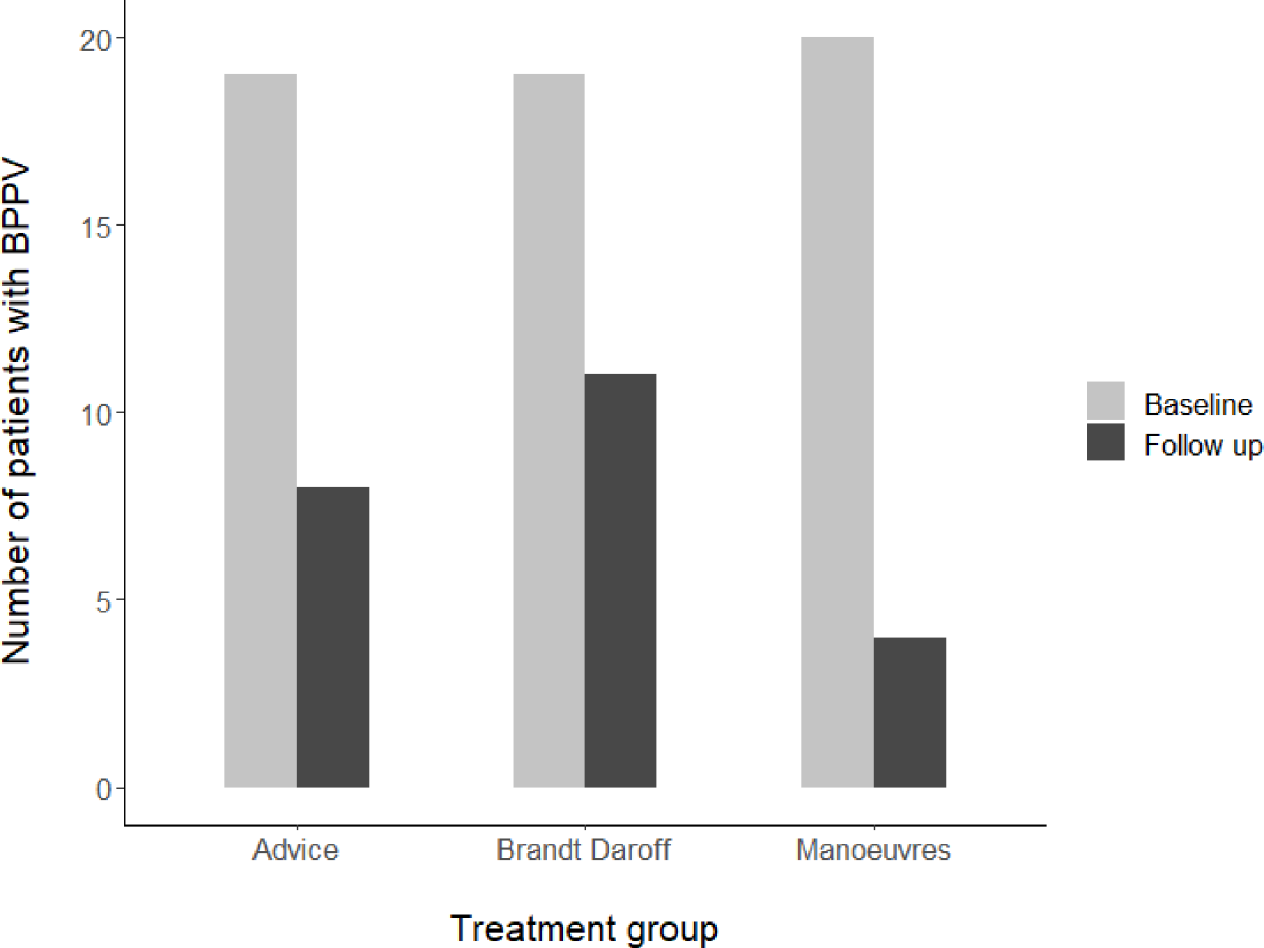
Number of patients with BPPV at baseline and 12-week follow up by treatment group

BPPV resolution at 12 weeks was noted in 35/58 (60%) of patients. Repositioning manoeuvres were more likely to lead to resolution of BPPV compared with both Brandt–Daroff and advice interventions, with an OR of 3.73 (95% CI 1.07 to 15.7; p=0.03) (figure 2). 10 patients experienced BPPV recurrence. Of these, 4/10 (40%) had a recurrence at 4 weeks and 6/10 (60%) at the end of the trial. 6/10 (60%) patients with recurrence were from the repositioning manoeuvre group. There were no differences in age between patients with and without recurrence; however, 7/10 (70%) initially presented with bilateral or mixed BPPV, and 8/10 (80%) had skull fractures.

Patients with BPPV were significantly more likely to have a skull fracture (p<0.001) than those without BPPV. Temporal bone fractures in particular were significantly associated with the presence of BPPV (multiple comparison correction: p=0.006). However, analyses revealed no significant relationships between the laterality of skull fracture and laterality of BPPV, the presence of BPPV and head injury severity (as measured by the Mayo classification system), or between the presence of BPPV and injury mechanism.

### Objective measures of static balance, gait and reported falls

**Figure 3:**
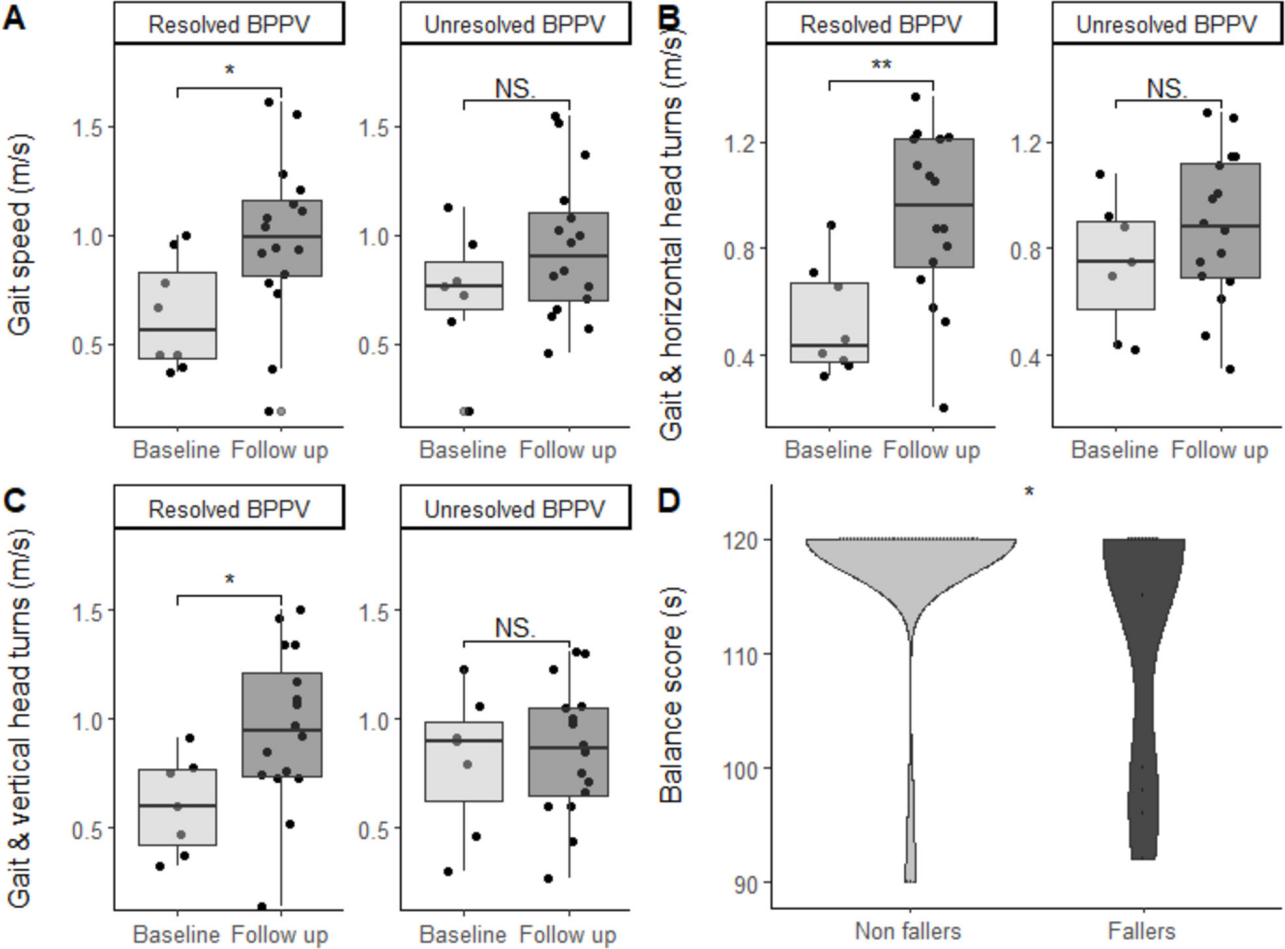
(A) Gait speed expressed in metres per second at baseline and follow-up in those with resolved (0.62 m/s to 0.93 m/s) and unresolved benign paroxysmal positional vertigo (BPPV) (0.72 m/s to 0.99 m/s). (B) Gait speed with horizontal head turns expressed in metres per second at baseline and follow-up in those with resolved (0.52 m/s to 0.92 m/s) and unresolved BPPV (0.74 m/s to 0.88 m/s). (C) Gait speed with vertical head movements expressed in metres per second in those with resolved (0.6 m/s to 0.96 m/s) and unresolved BPPV (0.8 m/s to 0.86 m/s). Baseline gait speed, gait speed with horizontal head turns and gait speed with vertical head movements were not significantly different in those with resolved and unresolved BPPV. (D) Balance scores as measured by the total score of the modified clinical test of sensory interaction in balance expressed in seconds in non-fallers and fallers (/120 s). The protocol involved quiet standing with feet together in four conditions: eyes open and closed on hard and soft surfaces. Abbreviation; NS, not significant. ^∗^=P<0.05 ^∗∗^=P<0.01.

Gait speed improved significantly from baseline to follow-up (p=0.04) in patients with resolved (0.61 m/s to 0.93 m/s) but not unresolved BPPV (figure 3). Gait speed with vertical head movements (p=0.02) and horizontal head turns (p*=*<0.001) also increased in only those with resolved BPPV. There were no statistically significant relationships between treatment group and change in gait speed.

We found an unexpectedly high rate of falls in our cohort despite the young average age of participants, with 11/58 (18.9%) patients reporting a fall during the trial. 7 of the 11 fallers (63.6%) had BPPV at the time of their falls. Falls were equally distributed among treatment groups. Age or gait speed did not differ between fallers and non-fallers; however, non-fallers were able to maintain static balance for a longer duration compared with fallers (95% CI 0.13 to 10.15 p=0.04) (figure 3).

### Self-reported measures

Patients with resolved BPPV demonstrated significant reductions in subjective dizziness scores compared with baseline (p*=*0.01), whereas dizziness scores remained unchanged in those without BPPV resolution (online supplemental figure 1). Similarly, Mann–Whitney U-test noted there was a significant different balance confidence (as reported by ABC scores) between baseline and follow-up in those with resolved BPPV (60% to 87%, W=44, p=0.04), while differences in those with unresolved BPPV did not show differences (75% to 76.2%, W=191, p*=*0.6). Health status as measured by the EQ-5D index score noted significant improvements in those with resolved BPPV (p=0.04) but no difference in those with unresolved BPPV (p*=*0.2). Mood scores improved significantly in those with both resolved and unresolved BPPV. No significant differences were noted in quality-of-life scores between those with resolved and unresolved BPPV. There were no effects of treatment group on any of the self-reported measures.

### Intervention monitoring and intervention fidelity

Time from assessment to discharge was short at 2 days (IQR 6.75). The median treatment time in the manoeuvre arm was 30 minutes (IQR 45) compared with 20 minutes in the Brandt–Daroff (IQR 22) and advice arms (IQR 8.75). These differences were not statistically significant. Participants in the manoeuvre group received on average 1.7 treatment sessions, generally spread over 2 or more days, and required on average 3.2 manoeuvres to achieve resolution acutely. There were no significant differences between patients with bilateral and unilateral BPPV in terms of the number of manoeuvres required for resolution. Clinician confidence was highest in the advice group (online supplemental table 4). Six treatment videos (>10% of all treatments) were obtained from all sites and treatment groups by a member of the research team who could be unblinded to treatment allocation without risk of bias. The experienced moderator noted treatments were performed in line with clinical practice guidelines (online supplemental table 5).

## Discussion

This is the first report of a prospective, randomised trial investigating therapist-led management of post-traumatic BPPV in acute settings. One of our feasibility aims was to determine the safety of undertaking therapy-led BPPV assessment and treatment in patients with acute TBI. We believe the results show that assessment and treatment procedures are safe in this population as (1) no *serious* adverse events were reported, (2) the frequency of adverse events is in line with previous rigorously conducted BPPV trials^21^ and (3) intervention monitoring criteria noted procedures were undertaken consistently and accurately between sites and therapists. This is an important finding given previous research has highlighted clinicians’ concerns regarding the safety of managing post-traumatic BPPV.^10^ Patient and therapist acceptability of assessment and treatment procedures, important for future implementation, will be reported separately.

In this study, BPPV was present in just over a third of acute TBI patients who were identified, eligible and agreeable to positional testing, a slightly lower figure than previously cited.^13^ This may be due to different recruitment criteria and a lower proportion of moderate-severe TBI in the present trial compared with other research.^13^ Based on previously published data, a higher rate of BPPV would be expected in a sample with a higher, overall TBI severity.^7^ Data from this study noted repositioning manoeuvres seemingly provided greater BPPV resolution. Interestingly, Brandt–Daroff exercises did not provide superior resolution compared with advice, perhaps due to the higher numbers of patients with more complex BPPV in the Brandt–Daroff group compared with the advice group. A larger, more definitive trial would be required to confirm the superior effectiveness of repositioning manoeuvres versus advice and Brandt–Daroff exercises.

We noted some interesting features of post-traumatic BPPV. Skull fracture and, in particular, temporal bone fracture, were significantly associated with the presence of BPPV, replicating findings from a previous single-centre study in TBI.^7^ The rate of bilateral BPPV in the present study is somewhat higher in comparison to previous subacute studies,^35 36^ a factor, alongside skull fracture, which was also linked to BPPV recurrence. The overall recurrence rate of BPPV in the present study is not dissimilar to previous research.^14 36^ However, recurrences noted in our data occurred during a much shorter follow-up period. Therefore, the optimal timing of treatment for patients at risk of recurrence remains unclear. Until this is established, we propose that the best clinical practice would be to closely monitor patients with bilateral or more complex BPPV and consider the provision of earlier follow-up. The mechanisms of BPPV recurrence are intriguing. An animal model of TBI showed persistent otoconial shedding over 12 weeks post-injury,^15^ while histopathological evidence noted degeneration of the utricle and semi-circular canals.^37^ If there are ongoing processes inimical for recovery of inner ear structure and function via inner ear hair cell regeneration or degeneration, then a single, acute repositioning treatment for patients with post-TBI BPPV may well be insufficient. We speculate the link noted between bilateral BPPV and/or skull fracture and recurrence is mediated by the amount of force sustained to the head at the time of injury. Higher forces may worsen inner ear ultrastructural damage and/or brain injury and, thus, lead to more protracted or less complete recovery.

Lastly, the lack of symptom reporting before and during diagnostic BPPV testing (ie, vestibular agnosia) in this study supports the need for objective, examination-based screening and treatment of post-traumatic BPPV, rather than traditional symptom-based screening. Indeed, symptomatic screening for BPPV found 6% BPPV rates^38^ versus 58% obtained by examination in moderate-to-severe TBI survivors in a rehabilitation setting, of whom less than 10% had vertigo symptoms.^39^ We previously showed that vestibular agnosia is linked to disrupted central brain circuits,^13 40^ which in turn is linked to TBI severity. Thus, differences in cohort TBI severity affect vestibular agnosia rates.

Our findings reveal that patients with acute post-traumatic BPPV had reduced gait speed compared with community-dwelling patients with idiopathic BPPV.^8^ There is currently little data on gait speed in patients with acute moderate-severe TBI, but it is likely there is a deleterious effect of BPPV on gait function that depends in part on the degree of brain injury. Gait speed is a known risk factor for falls in patients with^8^ and without BPPV.^41^ Compared with patients with idiopathic BPPV, post-traumatic BPPV patients are more likely to be at increased risk of falls, yet have less vertigo symptoms due to TBI-linked vestibular agnosia.^13^ In our study, almost 20% of TBI patients had a fall during the 12-week follow-up period, with 64% of fallers having active BPPV. The feasibility nature of the study did not allow us to investigate the underlying mechanisms of these falls; however, this would be a key aim of a future, larger trial. A recent systematic review and meta-analysis of community-dwelling patients with idiopathic BPPV noted repositioning manoeuvres reduced falls and improved gait speed.^8^ Similarly, data from the present study demonstrated repositioning manoeuvres were associated with BPPV resolution, increased gait speed and improved balance confidence. Given that secondary falls in TBI survivors carry significant morbidity and mortality,^6^ modifying fall risk, for example, by treating BPPV, could be considered critical.

The 2023 UK NICE acute head injury guidelines^11^ do not mention the need for BPPV assessment. However, in light of the findings that post-traumatic BPPV is highly prevalent,^2 7^ linked to falls (and thus morbidity and mortality) and not always associated with vertigo,^13^ we recommend a screening approach for BPPV in all those with acute TBI, but particularly patients with skull fracture or those with moderate-severe TBI. Previous studies have noted that trauma ward therapists^23^ and doctors working in emergency areas^42^ can be trained and mentored to manage BPPV. However, much implementation work remains to be undertaken in both trauma and accident and emergency settings for such skills to be embedded in practice. Given the link between post-traumatic BPPV and skull fracture noted in this, and a previous study,^7^ one approach could involve healthcare professionals who work in an accident and emergency setting assessing acute TBI patients for positional nystagmus and, if positive (regardless of cause), referring patients for a CT head scan and for a more complete vestibular neurology assessment. Further, specific work conducted in emergency areas is needed to validate this theory. A gold-standard approach might be for hospitalised acute traumatic brain injury patients to be assessed by a ward vestibular team (eg, comprising neurologist, therapist and vestibular scientist and other specialists with relevant expertise), since the complex balance problems affecting TBI patients typically combine peripheral and vestibular dysfunction, and are complicated by other neurological injury (brain, spine, muscle, peripheral nerve) and interactions with medication, including anti-epileptics^43^ and opiates.^44^

### Limitations

We recognise several limitations of our study. Despite the multi-centre design, the catchment population of our London (UK) trauma units limits generalisability. The self-reported dizziness measures used were predominantly designed for community-dwelling patients. An appropriate acute vestibular questionnaire is currently lacking and would be necessary for a future trial. Videonystagmography was not used to verify acute BPPV diagnosis; however, this is not mandatory as per the Barany Society criteria.^22^ It is possible that the overall BPPV frequency was underestimated as we did not routinely obtain videonystagmography; however, the therapists were trained by the research team, and videos were recorded of the therapists performing diagnostic and therapeutic manoeuvres, which confirmed diagnostic and treatment fidelity (see video). Despite this, some variation in the overall frequency of BPPV is expected given its link to TBI severity.^7^ Small variations in BPPV frequency between cohorts would not have affected the feasibility study’s aims and objectives. It is possible that some of our cohort who suffered a fall had undiagnosed pre-morbid idiopathic BPPV and, thus, may have been wrongly classified as having post-traumatic BPPV. A large proportion of patients who denied pre-morbid dizziness reduces this likelihood, although previous BPPV cannot be fully discounted due to the cognitive impact of TBI, for example, retrograde amnesia.

## Conclusions

This study found that clinical, bedside assessment and treatment of acute post-traumatic BPPV is safe and practical. Trial progression criteria were met, supporting the advancement to a multi-centre effectiveness trial. Repositioning manoeuvres demonstrated a superior resolution rate, but high rates of BPPV recurrence mean optimal timing of treatment remains unclear. BPPV resolution was associated with improved gait speed (itself linked to lower fall risk), while active BPPV was linked to falls. Thus, treating post-traumatic BPPV may modify fall risk.

## Data Availability

Data are available upon reasonable request. Data available on reasonable request.
