# Supplementary material for: Treating benign paroxysmal positional vertigo in acute traumatic brain injury: a prospective, randomised clinical trial assessing safety, feasibility, and efficacy
Source: BMJ Neurol Open. 2024 May 28;6(1):e000598. doi: 10.1136/bmjno-2023-000598 (PMC11138307; doi:10.1136/bmjno-2023-000598)
Supplement: Supplementary data [file bmjno-2023-000598supp001.pdf]

Supplementary information

Supplement 1 – Advice sheet

Title: A feasibility study investigating different interventions for the treatment of benign paroxysmal positional vertigo in acute traumatic brain injury patients.

Chief Investigator: Rebecca Smith, Imperial College, Division of Brain Sciences, Charing Cross Hospital Campus, W6 8RF. Tel: 02033117042

Why have I been given this sheet?
Following your head injury, you were diagnosed with a common form of dizziness called 'Benign Paroxysmal Positional Vertigo'. This is a condition where small 'crystals' in your inner ear become loose. Sometimes this can make you feel dizzy in certain positions and can cause problems with your balance.

You have agreed take part in the above research study which is investigating different treatments for this type of dizziness.

What is the treatment?
A therapist working on the ward will give you some advice about managing the dizziness. You can also keep this sheet which will explain the advice.
We will ask you to attend an outpatient appointment at the hospital to monitor your response to the treatment, at 4 and 12 weeks following your treatment.

<u>Advice for managing the dizziness:</u>

1. Try to move as 'normally as possible'. This means making sure you keep moving your head and body as much as possible. This includes trying to return to your daily activities as best as you can. Try to keep moving and try not to avoid difficult movements.

2. Give yourself a bit of time. Sometimes sitting on the edge of the bed for a few minutes before you stand up can help. You can also try also pausing for a few moments when you first stand up to get your balance, before you start to walk.

3. Try to maintain a good fluid intake. This means ensuring you are drinking plenty of water throughout the day.

Smith RM, *et al. BMJ Neurol Open* 2024; 6:e000598. doi: 10.1136/bmjno-2023-000598

4. Try and minimise your risk of falling over. This might mean having someone with you when you are walking around for safety, or simple things such as putting the light on at night time if you need to use the bathroom.

5. There are some factsheets about dizziness following head injury which have been written by the head injury charity, Headway. You may find these factsheets helpful:

   https://www.headway.org.uk/media/2787/balance-problems-and-dizziness-after-brain-injury-tips-and-coping-strategies-factsheet.pdf

   https://www.headway.org.uk/media/2786/balance-problems-and-dizziness-after-brain-injury-causes-and-treatment-factsheet.pdf

What if I have questions?
Please ask the therapist working on the ward to explain the advice to you if you do not understand. It might be helpful if a family member or carer is there too.

What should I do if I still feel dizzy when I get home?
The dizziness may go away on its own. If it doesn't keep following the advice above. We will send you an outpatient follow up appointment four weeks after you have been discharged from the hospital. We will assess you at the appointment to see if the dizziness has resolved.

Supplement 2

Table 1. List of outcome measures used in the trial and relevant time point

Outcome measure	Baseline	4 weeks	12 weeks
Subjective measures			
Dizziness handicap inventory	X	X	X
UCLA dizziness questionnaire	X	X	X
Activities specific balance confidence scale	X	X	X
Hospital anxiety and depression scale	X	X	X
EQ-5D	X	X	X
Montreal Cognitive Assessment	X	-	X
Glasgow Outcome Score Extended	-	X	X
Quality of life after Brain Injury	-	X	X
Work Quality Index	-	X	X
Objective measures			
Modified clinical test of sensory interaction in balance	X	X	X
Modified dynamic gait index	X	X	X

Table 2. Adverse events of vomiting across all three treatment groups

Patient ID	Group	BPPV type	Timing & medication
KC2207	Advice	Bilateral posterior canal	Post Epley at 12 week follow up
SM0607	Advice	Bilateral posterior canal	Post Dix Hallpike at 4 & 12 week follow-up<br><br>Required medication
SM0608	Manoeuvres	Bilateral posterior canal	Post Epley acutely
SG0706	Manoeuvres	Unilateral posterior canal	Post Epley acutely<br><br>Required medication
KC1406	Brandt Daroff	Mixed posterior and horizontal canal	Post Dix Hallpike at baseline

Smith RM, *et al. BMJ Neurol Open* 2024; 6:e000598. doi: 10.1136/bmjno-2023-000598

Table 3. Baseline characteristics of those with differing BPPV typologies. Mixed BPPV refers to posterior and horizontal canal BPPV.

Characteristic	Unilateral BPPV (n= 30)	Bilateral BPPV (n=23)	Mixed BPPV (n=5)
Sex (n, %)			
Male	26 (87%)	9 (39%)	4 (80%)
Female	4 (13%)	14 (61%)	1 (20%)
Age in years, mean (SD)	50.4 (19.61)	55.95 (15.59)	60.6 (14.67)
Injury details (n, %)			
Mechanism of Injury (RTA)	15 (50%)	5 (22%)	2 (40%)
Mechanism of Injury (Fall)	10 (33.3%)	13 (57%)	2 (40%)
Moderate-Severe TBI	21 (70%)	18 (78%)	4 (80%)
Skull fractures	22 (73%)	15 (65%)	3 (60%)
DHI, median (IQR)	18 (49)	38 (44)	27 (12)
FAC independent (n, %)	22	10	2
Gait speed m/s, mean (SD)	0.81 (0.23)	0.57 (0.26)	0.66 (0.41)

Abbreviation: RTA, Road traffic accident; DHI, Dizziness handicap inventory; FAC, Functional ambulation category

Figure 1. Boxplot noting Dizziness handicap inventory (DHI) scores in those with resolved and unresolved BPPV from baseline to 12 week follow up. Those with resolved BPPV had a median DHI of 28 (IQR 31.5) at baseline and a DHI of 8 (IQR 30) at follow up. Those with unresolved BPPV had a median DHI of 25 (IQR 44) at baseline and a DHI of 28 at follow up (IQR 29).

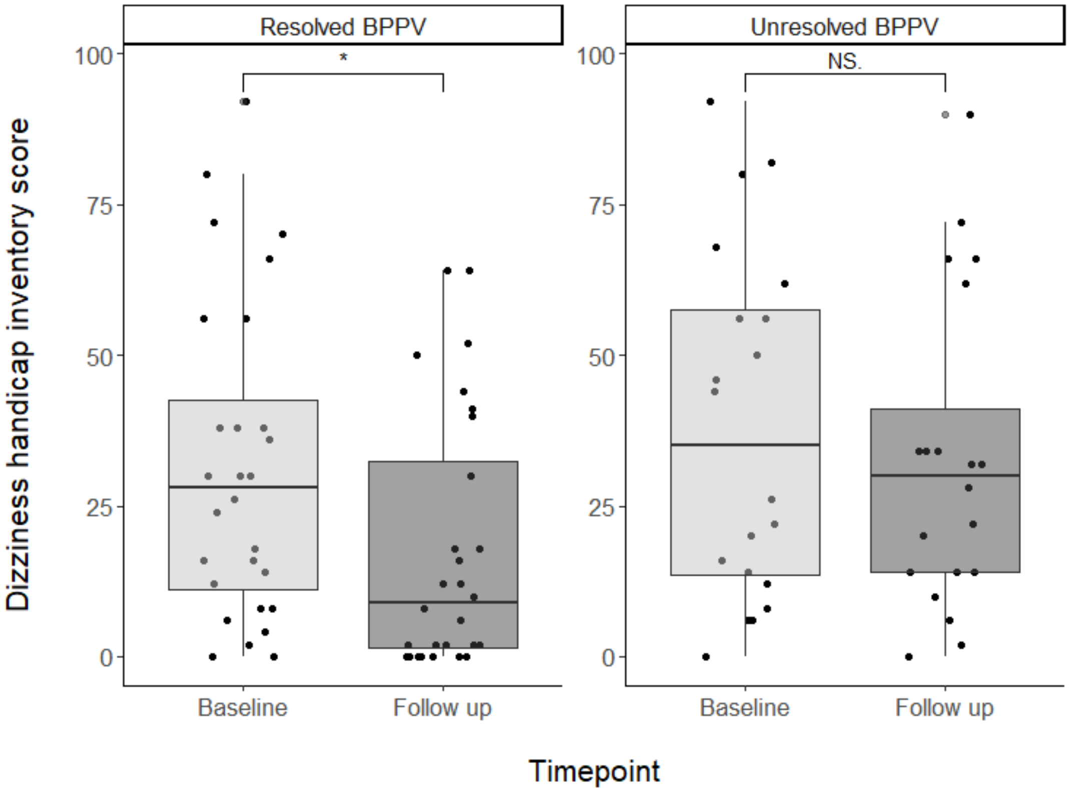

Abbreviation; NS, not significant. * = *P* <.05 ** = *P* <.01

Table 4. Intervention monitoring features

**Feature**	**Manoeuvres**	**Brandt Daroff**	**Advice**
Time in minutes, median (IQR)	30 (45)	20 (22)	20 (8.75)
Clinician confidence /10, mean (SD)	8.1 (1.5)	8.5 (1.06)	9.5 (0.7)
Number of manoeuvres required, mean (SD)	3.2 (2.5)	N/A	N/A
Adherence to exercises (%)	N/A	82	N/A

Table 5. Moderator scores across all sites and treatment groups

	**Assessment**	**Treatment**			
	Dix-Hallpike	Instructions	Accuracy	Speed	Overall
Mean score /10	8.6	8.8	8.4	9.6	9
